# NATIVE AMERICAN CAREGIVING PREFERENCES: HEARING THEIR VOICES

**DOI:** 10.1093/geroni/igae098.2127

**Published:** 2024-12-31

**Authors:** Pamela Monaghan-Geernaert

**Affiliations:** Northern State University, Aberdeen, South Dakota, United States

## Abstract

American Indian and Alaska Native elders have the shortest life expectancy of all non-white groups in the USA (CDC, 2022). Despite this disparity, Native elders experience higher rates of morbidity, often necessitating skilled care. Previous research has indicated that enhancing treatment for AI/AN elders must incorporate cultural considerations (Willging et al. 2021). This study utilized focus group data collected at the National Indian Council on Aging (NICOA) biennial conference for American Indian and Alaska Native elders. Elders from all Indian Health Service Units across the nation participated in the focus groups, sharing their desires for caregiving when they can no longer care for themselves. Thematic analysis of the data revealed that elders wish to have their voices heard and to play a role in guiding their own care to the best of their abilities. When external caregivers are necessary, elders prefer family members as their first choice, followed by tribal members, with culturally trained caregivers considered as a last resort. Additionally, elders expressed a desire for the incorporation of Native teachings, storytelling, and traditional medicines, such as herbal remedies, into their care. They also emphasized the importance of streamlining the care process between Indian Health Services (IHS), tribes, and families and removing barriers to ensure respective care during periods of illness and frailty. As the Native elderly population continues to age, it is crucial that we actively listen to their stories and preferences regarding elderly care.

